# Results and challenges of *Cytochrome P450 2D6* (*CYP2D6*) testing in an ethnically diverse South Florida population

**DOI:** 10.1002/mgg3.922

**Published:** 2019-08-07

**Authors:** Daria Salyakina, Sharmeen Roy, Weize Wang, Mailin Oliva, Rohan Akhouri, Ileana Sotto, Nicole Mulas, Rafaela Solano, José R. Fernández, Stephanie Sanchez, Uzma Shamshad, Chad Perlyn, Jennifer McCafferty‐Fernandez

**Affiliations:** ^1^ Research Institute Nicklaus Children’s Hospital Miami Florida; ^2^ Department of Nutrition Sciences University of Alabama at Birmingham Birmingham Alabama; ^3^ Herbert Wertheim College of Medicine Florida International University Miami Florida

**Keywords:** CYP2D6, ethnicity, Hispanic, intermediate metabolizers, normal metabolizers, pharmacogenetics, poor metabolizers, race, ultrarapid metabolizers

## Abstract

**Background:**

This study focuses on the implementation of *CYP2D6* genetic test profiling and the challenges associated with using standard pharmacogenetics panels in a diverse South Florida population.

**Methods:**

A total of 413 participants were recruited to participate in this study through Nicklaus Children's Hospital. Buccal swabs were collected and tested using an extended *CYP2D6* panel including 22 alleles. Phenotype, genotype, and allelic frequencies were compared among different racial and ethnic groups.

**Results:**

The majority of participants (75.0%) self‐identified as Hispanics. Four alleles, CYP2D6*4, *17, *41, and *2A, showed a statistically significant difference between White Hispanics and Black Non‐Hispanics. Aggregate frequency of all alleles with decreased function varied between 2.8% and 50.0% in different racial and ethnic groups. Additionally, rare allele combinations were observed in this South Florida cohort.

**Conclusions:**

The heterogeneity among Hispanic groups demonstrated in previous literature and by this study reflects the complexity of ethnicity and suggests that a more granular categorization is needed, one based on ancestry and migration history rather than primary language. Overall, we have determined that there are statistically significant differences in *CYP2D6* allele frequencies in the distinct racial and ethnic populations of South Florida, demonstrating a unique genetic makeup within South Florida. However, overall, the frequencies of Poor Metabolizer, Normal Metabolizer, Intermediate Metabolizer, and Ultrarapid Metabolizer did not differ between racial and ethnic groups at a statistically significant level.

## 1 INTRODUCTION

*Cytochrome P450 2D6 *(*CYP2D6*) is an enzyme metabolizing approximately 25% of therapeutic drugs, including antidepressants, antipsychotics, analgesics, antitussives, beta‐adrenergic blocking agents, antiarrhythmics, and antiemetics (NIH, 2017a). This drug‐metabolizing enzyme is encoded by a highly polymorphic *CYP2D6* gene with more than 100 variant alleles located on chromosome 22q13.1 (Daly et al., 1996; NIH, 2017a) (OMIM #608902, NM_001195406). Each *CYP2D6* allele (or haplotype) is defined by a specific profile of single‐nucleotide polymorphisms (SNPs), insertions, deletions, duplications, and multiplications (Crews, 2014). As allelic variants influence protein expression and activity, *CYP2D6* polymorphisms impact the enzyme's functional capacity to metabolize therapeutic drugs. *CYP2D6* alleles are phenotypically defined by their expected influence on overall *CYP2D6* enzymatic activity, such as no function, decreased function, normal function, increased function, unknown, and uncertain function (Caudle, 2017). An individual's *CYP2D6* diplotype, or their combination of maternal and paternal alleles, comprehensively determines *CYP2D6* metabolic activity as poor metabolizers (PMs), intermediate metabolizers (IMs), normal metabolizers (NMs), or ultrarapid metabolizers (UMs) (NIH, 2017a). An UM phenotype is a result of gene duplication or multiplication which occurs from inheriting more than two copies of the fully functional *CYP2D6* alleles (UM) (Crews, 2014).

The efficacy and toxicity of a therapeutic drug metabolized by *CYP2D6* can differ from person‐to‐person depending on an individual's *CYP2D6* genotype and phenotype. One example is codeine, an opioid analgesic indicated to relieve mild to moderate pain through codeine metabolism and morphine activation by *CYP2D6* enzymes (NIH, 2017b). Data show that *CYP2D6* PMs demonstrate significantly lower morphine serum concentrations and analgesia in comparison to NMs who receive identical doses of codeine (Eckhardt et al., 1998). Meanwhile, *CYP2D6* UMs exhibit an increased conversion of codeine to morphine in comparison to NMs, which can result in toxic concentrations of morphine and life‐threatening adverse reactions (Ciszkowski, Madadi, Phillips, Lauwers, & Koren, 2009; Dalén, Frengell, Dahl, & Sjöqvist, 1997; Gasche, 2004). For this reason, it is vital to understand *CYP2D6* pharmacogenetics and how *CYP2D6* polymorphisms influence a patient's favorable or adverse clinical responses to drugs metabolized by *CYP2D6* enzymes (NIH, 2016).

Based on the U.S. census estimates in 2016, 67.7% of Miami‐Dade residents identified as Hispanic or Latino compared to 17.8% nationally (U.S. Census Bureau, 2016). Given that the *CYP2D6* allele, genotype, and phenotype frequencies may differ substantially among ethnic and racial groups*,* pharmacogenetic testing may be used to personalize and improve treatment for diverse patient populations (NIH, 2017; Owusu‐Obeng et al., 2014). Studies have demonstrated that certain Hispanic groups have *CYP2D6* alleles that are rare in non‐Hispanic populations (Bernard, 2006; Casner, 2005; Gaedigk, 2010; Luo, Gaedigk, Aloumanis, & Wan, 2005). However, current pharmacogenetics research focuses primarily on White Non‐Hispanics (Claudio‐Campos, Duconge, Cadilla, & Ruaño, 2015; Ortega & Meyers, 2014; Ramos, Callier, & Rotimi, 2012), which may lead to a misrepresentation of allelic frequency estimates in highly diverse Hispanic or Latino populations, such as those in South Florida.

The genotyping of *CYP2D6* involves analysis of a select number of known SNPs and genetic variants. However, the number of screened variants is inconsistent across laboratories, ranging from 3 to 35 alleles (Flores‐Angulo, 2015; Gaedigk, Bradford, Marcucci, & Leeder, 2002; Gaedigk et al., 1999; Kohlrausch, 2009; Leathart et al., 1998; López, Guerrero, Jung‐Cook, & Alonso, 2005). The inconsistencies in results are due to laboratories differing in test design, including what alleles are interrogated and how the alleles are identified. This leaves many alleles untested and undetected, which may return false‐negative results (Bell, 2017; Gaedigk et al., 1999).

Our study focused on the unique *CYP2D6* genetic profile of the South Florida population. We examined the allele and phenotype frequencies among different combinations of races and ethnicities. Due to a lack of diversity in the currently available pharmacogenetics research and data, we hypothesized that these differences would be statistically significant in the South Florida population.

## 2 MATERIALS AND METHODS

### 2.1 Ethical compliance

Western Institutional Review Board approved the study and all participants and their caregivers provided consent/assent when appropriate.

Patients of Nicklaus Children's Hospital were recruited to participate in this study (*N* = 413). Most participants enrolled at the end of their visit with the neurology, plastic surgery, or hematology/oncology departments. Patients and family members attending the hospital's Cancer Center Mini Relay for Life were also introduced to the study and invited to participate. All patients had to be between the ages of 1–21 years and reside in the South Florida area to be eligible for the study.

Participants were asked to provide their race as either White, Black, Asian, or Mixed, and categorized their ethnicity as Hispanic or Non‐Hispanic. There were 37 participants (8.9%) who refused to provide this information. These patients were included in the analysis of total frequencies of phenotypes. Buccal swabs were collected for DNA extraction. All samples were genotyped at the Genelex^®^ Corporation (Genelex Corporation) using an extended *CYP2D6* (OMIM #608902, NM_001195406) panel with enough sensitivity to capture over 93.0%–97.0% of the low‐frequency PM phenotypes. Genelex^®^ was selected based on several factors including clinical decision support, turnaround time, breadth of the panel, and cost. Twenty‐two alleles were included in the panel: CYP2D6 *1, *2, *2A, *3, *4, *5, *6, *7, *8, *9, *10, *11, *12, *14A, *15, *17, *19, *20, *29, *35, *36, and *41.

All participants and their primary caregivers if requested were mailed a copy of their individual phenotype/genotype results. The patients were also offered a consultation with a genetic counselor if they wanted a detailed explanation of the study results.

Subsequently, genotype results were analyzed. Allelic frequencies were assessed for statistically significant differences at a 95% confidence interval (CI) for proportions. CI was calculated as *p* ± 1.96 × SE, where SE = sqrt(*p* × (1 − *p*)/*n*); *p* is the proportion of an allelic variant or a phenotype in a given race and ethnicity group; n is the total number of patients in the same group. If 95% CI did not overlap, the difference was determined to be statistically significant. All statistical analysis was performed in R. Of note, the interpretation of activity scores by Genelex^®^ for PMs, IMs, NMs, and UMs differed from that of Clinical Pharmacogenetics Implementation Consortium (CPIC) guidelines. CPIC definitions were used in this study, namely, activity scores of 1.0–2.0 were considered NM.

## 3 RESULTS

**Figure 1:**
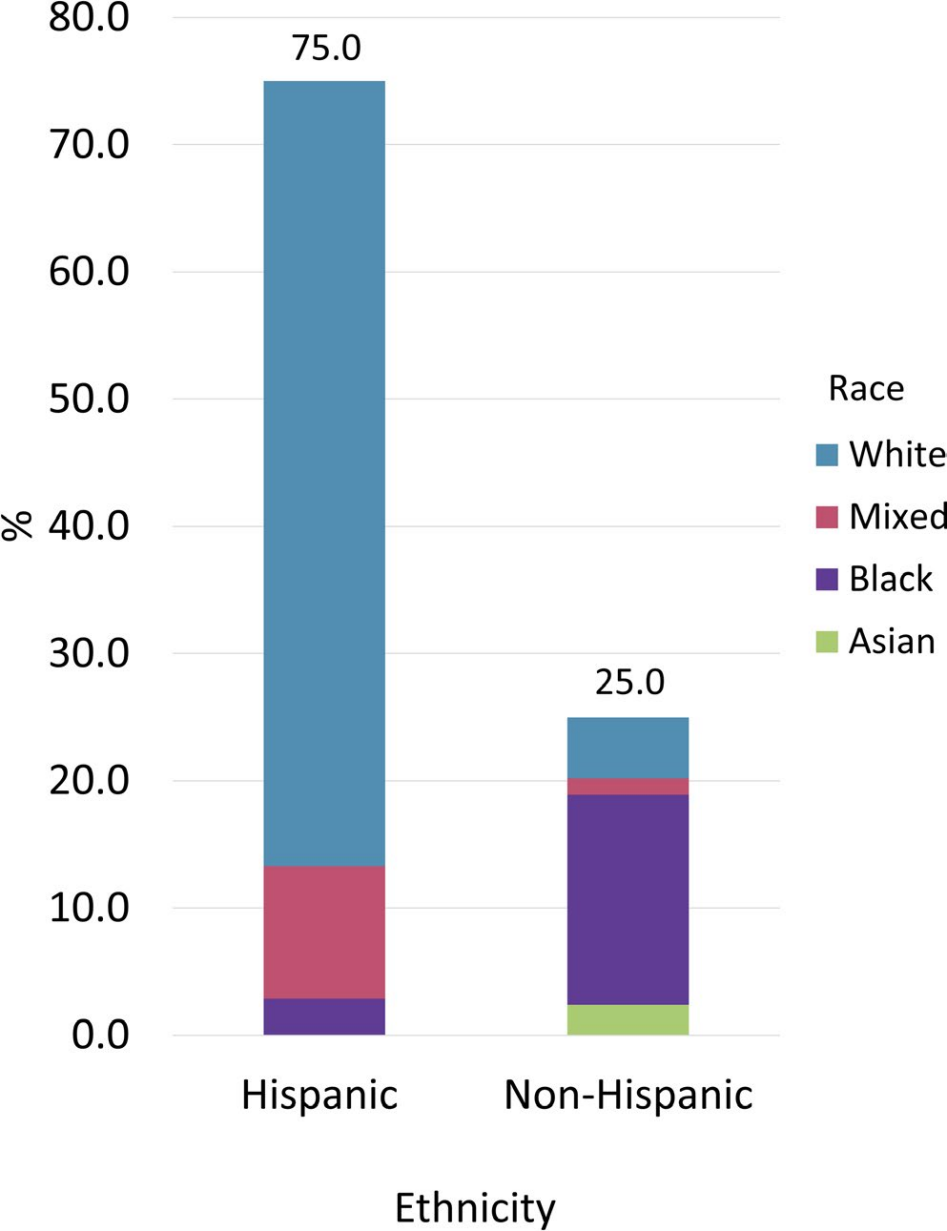
Study Participants' Ethnicity and Race (*N* = 376 nonmissing values). 75% identified Hispanic as their ethnicity. Most of the participants (62%) were White Hispanic (WH) followed by Black Non‐Hispanic (BNH) (16%)

**Figure 2:**
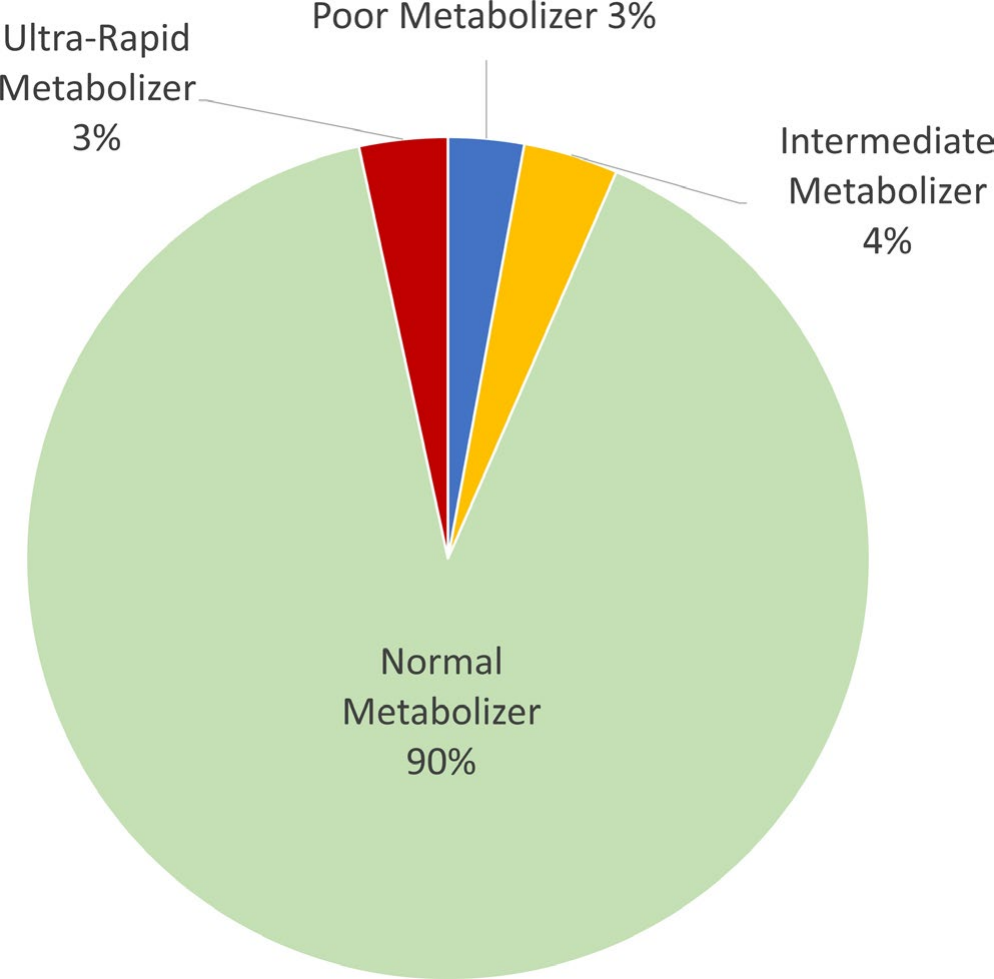
Phenotypes of 413 participants. OMIM gene ID: 608902, NM_001195406

Of the 413 individuals enrolled in the study, 376 provided information on their race and ethnicity, 75.0% identified as Hispanic, and 19.0%, 2.0%, and 11.0% identified their race as Black, Asian, and Mixed, respectively (Figure 1, Table 1). Most of the participants (62.0%) were White Hispanic (WH) followed by Black Non‐Hispanic (BNH) (16.0%). The most common phenotype in this cohort was NM (90%), followed by IM (4.0%) (Figure 2). The UM and PM phenotypes were equally represented at 3.0%. Of the 22 *CYP2D6* alleles examined, 14 were identified in the South Florida population: CYP2D6*1, *2, *2A, *3, *4, *5, *6, *9, *10, *17, *29, *35, *36, and *41. Eight alleles from the panel were not present: CYP2D6*7, *8, *11, *12, *14A, *15, *19, and *20.

**Table 1:** CYP2D6 phenotype frequencies grouped by race and ethnicity

Frequency	White Non‐Hispanic	White Hispanic	Mixed Non‐Hispanic	Mixed Hispanic	Black Non‐Hispanic	Black Hispanic	Asian Non‐Hispanic
Poor metabolizer	1	10	0	0	0	0	0
Intermediate metabolizer	0	9	0	0	3	0	0
Normal metabolizer	17	203	5	38	56	11	9
UltraRapid metabolizer	0	10	0	1	3	0	0
Total	18	232	5	39	62	11	9

Note

OMIM gene ID: 608902, NM_001195406.

**Figure 3:**
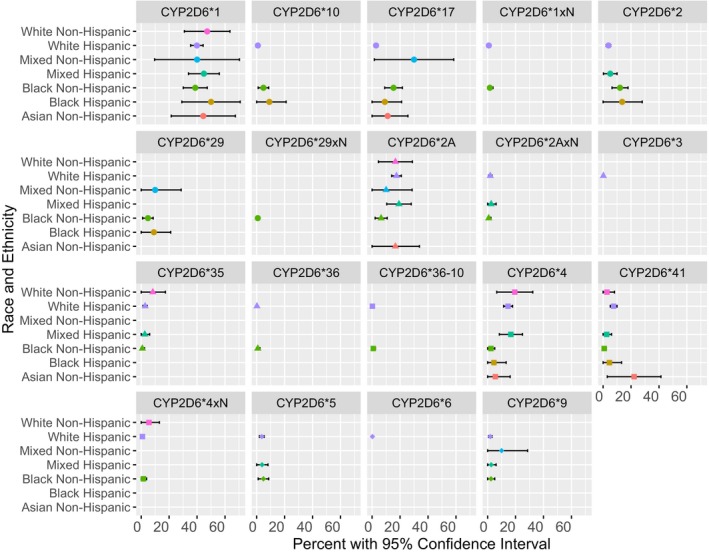
CYP2D6 allele frequencies grouped by race and ethnicity and enzyme activity categories with corresponding 95% CI. The biggest difference in allele frequencies among the “poor metabolizers” was observed for the *4 allele, varying between 2.4% and 14.7% in Black Non‐Hispanic and White Hispanic respectively. OMIM gene ID: 608902, NM_001195406

In total, 10 SNPs and 10 deletions/duplications were detected, showing variable allele frequencies among different race/ethnic groups (Figure 3). There were significant differences in frequencies among participants depending on their race and ethnicity for four alleles: CYP2D6*4, *17, *41, and *2A (Figure 3). The occurrence of a no‐function allele *4, was more prevalent in WH (14.7%) than in BNH (2.4%). As a result, PM genotype combinations were only detected in individuals self‐identifying as White.

Aggregate frequency of all decreased function alleles varied between 2.8% in White Non‐Hispanic participants and 50.0% in Mixed Non‐Hispanics. BNH subjects when compared to WH had a significantly higher frequency of CYP2D6*17 (15.3% vs. 2.8%), while CYP2D6*41 showed a significant difference in the opposite direction (0.8% vs. 7.5%). (Figure 3).

Furthermore, in our study, the normal function CYP2D6*2A allele was significantly more frequent in the WH population (17.5%) than in the BNH (6.5%).

Finally, a rare allele combination and a unique duplication were observed in this South Florida cohort. Specifically, we found a BNH individual with four copies of normal function including CYP2D6*1, *2A, and two *2‐like alleles. Since no deleterious mutations were detected, it is assumed that all four alleles are active, and the phenotype would be expected to be UM. In addition, there was one BNH (0.8%) and one WH (0.2%) study participant with the rare tandem allele *36‐10.

Overall, we have determined that there are statistically significant differences in *CYP2D6* allele frequencies in the distinct racial and ethnic populations of South Florida, demonstrating a unique genetic makeup within South Florida. However, overall, frequencies of PM, NM, IM, and UM did not differ between racial and ethnic groups at a statistically significant level (Table 1). The power to detect allele frequency differences between WH and BNH in our study was 90%, but to detect differences between other less represented racial and ethnic groups dropped below 70%.

## 4 DISCUSSION

The distribution of phenotypes in this study was similar to what was previously described in literature; 4.0% IM, 3.0% PM, 3.0% UM, and 90.0% NM versus 2.8%, 1.9%, 4.6%, and 92.3%, respectively (Flores‐Angulo, 2015). However, the distribution of allele frequencies significantly varied between ethnic and racial groups. Namely, alleles CYP2D6*2A, *4, *17, and *41 showed prominent differences among WH and BNH.

The nonfunctional allele CYP2D6*4 was frequent in WH (14.7%), but not in BNH (2.4%), aligning with other studies on WH participants from Cuba, Nicaragua, and Venezuela (Flores‐Angulo, 2015; Griman, 2012; Kohlrausch, 2009). Furthermore, a meta‐analysis of allele frequency studies on healthy Puerto Ricans determined that their *4 frequency was similar to White Non‐Hispanics (Bernard, 2006). The *4 allelic frequencies of a Mexican‐American population revealed a somewhat lower frequency of 10% (Luo et al., 2005). Lastly, the *4 allele was less frequent in South Florida BNH compared to other BNH populations described in the literature (2.4% vs. 5.4%–7.8%) (Bradford, Gaedigk, & Leeder, 1998; Cai, 2006; Gaedigk et al., 2002; Leathart et al., 1998; US Census Bureau, 2010a; Wan et al., 2001).

Furthermore, BNHs in this study had a greater frequency of decreased function allele CYP2D6*17 (15.3%) compared to WHs (2.8%). These findings also align with reports from other studies on similar populations (Agúndez, Ramirez, Hernandez, Llerena, & Benítez, 1997; Bradford et al., 1998; Cai, 2006; Gaedigk et al., 2002; Isaza, Henao, López, & Cacabelos, 2000; Kohlrausch, 2009; Leathart et al., 1998; Llerena et al., 2012; Wan et al., 2001). However, other in‐depth studies are demonstrating significant variation among Hispanics. Namely, one study compared the presence of *17 between Cuban Mestizos, Nicaraguan Mestizos, and White Cubans. The allele was most prevalent in the Cuban Mestizos (10.2%). Meanwhile, none of the Nicaraguan Mestizos had *17, but 2.7% of the White Cuban population did (Llerena et al., 2012). Another study conducted in Costa Rica analyzed three different ethnic groups: Mestizo, Amerindian, and Afro‐Caribbean, and demonstrated that a greater proportion of the Afro‐Caribbean group (18.4%) had *17. The Mestizo and Amerindian populations also had *17 but to a lesser extent (1.8% and 2.2% respectively) (Céspedes‐Garro, 2014).

Another allele with decreased function showing significant differences between South Florida's racial and ethnic groups was CYP2D6*41. It was found in 7.5% of WH and only 0.8% of BNH populations. In comparison, a Spanish study reported a frequency of 3.8% in WH (Fernández‐Santander, 2010) and a study on Mexican‐Americans found a frequency of 9.5% (Luo et al., 2005). Furthermore, the reported frequencies of *41 in BNH populations markedly vary (1.8%–14.4%) (Cai, 2006; Gaedigk, 2005). Notably, alleles *41 and *2A were not distinguished until recently. Specifically, *2A involves a −1584C>G substitution while *41 involves a 2988G>A polymorphism (Daly et al., 1996). Therefore, before the 2988G>A SNP was characterized as a unique marker identifying *41, the sole method of differentiating *41 from *2A was by testing for the absence of SNP 1584C>G (Kennedy, 2008). Because of their similarity, not all prior studies tested for *41 and may have inadvertently reported larger frequencies of *2A in the tested populations. These issues have challenged the ability of researchers to accurately report *2A frequencies until recently (Blake, 2007; Cai, 2006; Gaedigk et al., 1999; Gaedigk, 2005, 2008; Griman, 2012; Kennedy, 2004, 2008).

This study is the first to identify a CYP2D6*36‐10 allele in a WH participant. The tandem allele *36‐10 is prevalent in Japanese populations (24.2%–26.7%), but rarely detected among African‐Americans (0.4%), and not reported in White populations (Hosono, 2009; Kiyotani, 2010; Soyama et al., 2004; Soyama, 2006). There are insufficient data on Hispanics or Latinos. The combination of a decreased function allele *10 with the no‐function allele *36 is thought to contribute to a PM phenotype (Gaedigk, Bradford, Alander, & Leeder, 2006; Kiyotani, 2010). Notably, *10 has conflicting data on several Puerto Rican populations. One study stated that *10 was found less frequently than in Caucasian populations, while another reported that Puerto Ricans have a *10 frequency similar to Caucasians (Claudio‐Campos, Orengo‐Mercado, et al., 2015).

Our research findings describe more accurate representations of CYP2D6*2, *2A, *17, and *41 allele frequencies in South Florida WH and BNH populations, and also identify a rare *36‐10 allele in a WH, as well a unique *2A duplication that has not been well‐described in Hispanic populations. Finally, *CYP2D6* test results revealed that the PM phenotype was only found in White individuals, which could be potentially explained by our low number of non‐White study participants. These results highlight the need for data on a larger number of non‐White populations and ethnic minorities.

In general, the frequency data on CYP2D6 *4, *17, *41, and *2A in diverse populations is limited. Therefore, there is a need to better characterize the *CYP2D6* genotype and phenotype variability in Hispanics and Latinos.

In addition to these frequent alleles, there may be others that are unique to Hispanics and Latinos that were not included in the study panel. For example, there have been studies among Latin American, Central American, and Hispanic countries that have provided insight into unique allelic variants. One such study involved Mexican Mestizos, Mexican individuals with a mixed European and American Indian background. They discovered a rare variant CYP2D6*82 at a frequency of 2.1% (Kiyotani, 2010). The functionality of CYP2D6*82 is currently unknown but the origin was suggested to be American Indian. Another study on a Mexican Mestizo population identified the normally functioning allele *53 in 0.5% of the sample (Contreras, 2011). Studies have also identified rare no‐function CYP2D6*31 and *40 in several Hispanic populations. For instance, the allele *31, associated with PMs, was discovered in two Spaniards and two Puerto Ricans (Gaedigk, 2010). Another genotyping study on a Puerto Rican cohort noted two individuals: one who had *31 and one with *40 (Gaedigk, 2010). Approximately 2.1%, 3.7%, and 0.6% of the Hispanic residents of Miami‐Dade County reported themselves in the 2010 Census as Mexican, Puerto Rican, and Spanish, respectively (US Census Bureau, 2010b). Allele *31 was not tested in this study, but it would be beneficial to include it in future pharmacogenomic panels for the South Florida population.

Although there were significant variations in allelic frequencies, no statistically significant differences in phenotype frequency were observed among the South Florida cohort evaluated in this study. This may be a result of an insufficient sample size of non‐Hispanic and non‐White cohorts and future larger studies are needed to prove this hypothesis. Past studies demonstrated that the frequency of *CYP2D6* phenotypes among several Hispanic and Latino groups differs from that of White non‐Hispanics across the Americas. A retrospective review of phenotype frequencies among ethnic groups found that the frequency of PMs in non‐Hispanic Whites from the Americas is 7.7%, while the frequency of PMs among Colombians, Mexicans, Panamanians, and Nicaraguans is markedly lower (6.6%, 3.2%, 2.2%–4.4%, and 3.6% respectively) (Bernard, 2006). At the same time, Mexican Mestizo had a frequency of PM phenotype similar to the white Spanish population (10%) (López et al., 2005).

The review of UM phenotype frequencies also demonstrated ethnicity‐based differences. Hispanic UMs were less common when compared to white American UMs (1.7% vs. 4.3%) (Bernard, 2006). The heterogeneity among Hispanic groups demonstrated in previous literature and by this study reflects the complexity of ethnicity and suggests that a more granular categorization is needed, one based on ancestry and migration history rather than primary language. These results prove once again that Hispanic ethnicity is not homogeneous, but rather is a mix of various populations with very diverse genetic backgrounds.

This study has a few limitations. WH and BNH were the two largest population groups in the study. It is therefore not surprising that the only statistically significant differences found were between the two ethnic groups with the largest sample sizes. It is possible that there are additional frequency differences among other groups in this population, but this study is underpowered to detect them. In addition, the race and ethnicity used in this study were based on self‐reported records by the study participants. These records may be imprecise and biased. Future studies should include design and resources to allow for genetic confirmation or verification of perceived ancestry.

Overall, our findings suggest that in predominantly Hispanic or Latino populations, the assumption that allelic and phenotype distributions are similar to less diverse populations may be inaccurate. As a result, pharmacogenetic panels designed for less diverse populations may not be appropriate for Hispanic populations. If the subject of a test has an allele that was not included in the panel, they risk improper classification of their *CYP2D6* function. For example, CYP2D6*31, *49, *50, *54, *55, *59, and *84 all translate to decreased or no function, however, they were not tested in this study and are not included in most commercial panels. It is currently not known whether these alleles are prevalent in Hispanics or Latinos. If a patient had any of these alleles, they would be incorrectly classified as NM. That could explain the markedly lower rates of PM previously reported in Hispanics (Bernard, 2006). Thus, pharmacogenetic test results would be more useful and accurate if laboratories sequenced the entire *CYP2D6* gene, rather than selected polymorphisms. As a whole, sequencing would also provide a better depiction of the allelic variation within underrepresented communities (Barron et al., 2016; Bernard, 2006; Claudio‐Campos, Orengo‐Mercado, et al., 2015; Ortega & Meyers, 2014). Importantly, minority groups are predicted to become the majority by 2044, with a quarter being Hispanic (Colby & Ortman, 2015). As the nation's diversity grows, it is critical that the available pharmacogenetic tests reflect the heterogeneity of the population.

## CONFLICT OF INTERESTS

None declared.
